# Experiencing improved assessment and control of pain in end‐of‐life care when using the Abbey Pain Scale systematically

**DOI:** 10.1002/nop2.566

**Published:** 2020-07-23

**Authors:** Carola Ludvigsson, Ulf Isaksson, Senada Hajdarevic

**Affiliations:** ^1^ Department of Nursing Umeå University Umeå Sweden; ^2^ Arctic Research Centre Umeå University Umeå Sweden

**Keywords:** end‐of‐life care, nurses, nursing, pain, terminal care

## Abstract

**Aim:**

To describe staff's reflections on aspects influencing pain assessment at end‐of‐life (EoL) care in nursing homes before and after the implementation of the Abbey Pain Scale (APS).

**Background:**

People with cognitive impairment in the EoL care often suffer from underdiagnosed and undertreated pain due to the lack of knowledge and guidelines for systematic pain assessment.

**Methods:**

Semi‐structured focus group interviews were conducted and analysed using qualitative content analysis.

**Results:**

The staff described their experiences before the implementation of APS as *striving to achieve control of pain by trusting in themselves and the team,* while the experiences after the implementation of APS were described as *improving symptom control with remaining weak confidence in the team*.

**Conclusions:**

Implementation of APS was experienced as improving systematic pain assessment. Efforts to establish clear routines and improve confidence in the care team would be prioritized to optimize pain assessment and pain relief in EoL care.

## 1 INTRODUCTION

People with cognitive impairment at the end‐of‐life care (EoL care) often have their pain underdiagnosed and undertreated due to the lack of knowledge and lack of guidelines for systematic pain assessment. According to recommendations from The National Board of Health and Welfar [Socialstyrelsen] (2013a), systematic pain assessments in EoL care should have high priority in providing optimal treatment of pain.

In Sweden, about 90,000 people die annually and it is estimated that about 80% of these would benefit from palliative care (The Swedish Palliative register, 2018). According to Statistics Sweden (2018), the expected number of people older than 65 years in 2030 will increase, especially those over 80 years. This indicates an increasing need of further development of palliative care. Good palliative care at the EoL means symptom relief of physical and mental pain, relief of social and existential problems, a multi‐professional collaboration in taking care of people, as well as good communication and relationship with the patient and relatives (The National Board of Health & Welfar [Socialstyrelsen], 2013a, 2013b).

## 2 BACKGROUND

Many people living in nursing homes for the elders in Sweden have dementia and cognitive impairment (Lovenheim, Sandman, Kallin, Karlsson, & Gustafson, 2008). That mirrors the high demands on nurses to perform qualified palliative care in EoL (The National Board of Health & Welfar [Socialstyrelsen], 2013a). Furthermore, many older people in EoL may have difficulties expressing pain verbally and instead react with anxiety, aggression, fatigue or nausea (Fürst, Lindqvist, & Tishelman, 2012). It has been described as challenging to interpret and assess whether and how much people with cognitive impairment, who cannot express pain verbally, suffer from pain and receive a good care (Cunnigham, McClean, & Kelly, 2010; Givard & Poulsen, 2013; Jansen et al., 2017; Monroe, Carte, Feldt, Tolley, & Cowan, 2012). Thus, optimal pain relief and pain assessment in EoL care can be difficult, especially regarding difficulties of detecting the cause of symptoms that can vary and be expressed in different ways by different people (Jansen et al., 2017).

A lack of systematic use of pain assessment among people with cognitive impairment together with their difficulties to verbally express pain (Cunnigham et al., 2010; Herr & Ersek, 2009; Monroe et al., 2012; Schulman‐Green et al., 2010) may lead to an under‐treatment of pain relief (Burns & McIlfatrick, 2015; Fuchs‐Lacelle, Hadjistavropoilos, & Lix, 2008; Lints‐Martindale, Hadjistavropulos, Lix, & Thorpe, 2012; McAuliffe, Nay, O'Donell, & Fetherstonhaugh, 2009; Tsai, Jeong, & Hunter, 2018). According to the European Association for Palliative Care (EAPC) (van der Steen et al., 2014), symptoms indicating pain among people with dementia in EoL care should be assessed by using validated pain assessment tools. However, today there are few guidelines and standards regarding pain assessment for these people (Ni Thuathail & Welford, 2011; Sampson et al., 2015; Tapp et al., 2019).

The knowledge among healthcare personnel (hereafter called staff) about individuals, their reactions and habits, as well as the staff's ability to recognize pain and use instruments to identify symptoms of pain, can reduce suboptimal treatment of pain and increase the quality of life for people at the EoL (Brorson, Plymoth, Örmon, & Bolmsjö, 2014; Herr et al., 2006; Zwakhalen, Hamers, & Berger, 2007; Zwakhalen, Hamers, Peijnenburg, & Berger, 2007). Hence, good communication among staff and relatives can be crucial for the improvement of pain assessment and treatment of people with cognitive impairment in EoL care (Jansen et al., 2017; Tarter, Demiris, Pike, Washington, & Parker Oliver, 2016; Zwakhalen, Hamers, & Berger, 2007; Zwakhalen, Hamers, Peijnenburg, et al., 2007). Research shows that pain assessment in people with cognitive impairment is not an ordinary implemented routine and staff experience these assessments as difficult (Gropelli & Sharer, 2013).

People with dementia and cognitive impairment may not obtain the same optimal care at the EoL as others do, which unnecessarily places them at risk of suffering due to non‐optimal pain management. Nurses meeting these people have the essential role of performing optimal pain management (Afzal, Buhagiar, Flood, & Cosgrave, 2010; Brorson et al., 2014; Gilmore‐Bykovskyi & Bowers, 2013; Newton, Reeves, West, & Schofield, 2014; Shega, Hougham, Stocking, Cox‐Hayley, & Sachs, 2008). There is not much research that focuses on the staff's experience of pain assessment among people with dementia and cognitive impairment at the EoL, especially in a Swedish context. Still, less than half of those who died in nursing homes in Sweden in 2018 have had their pain assessed with validated assessment instruments (The Swedish Palliative register, 2018). It is important to explore the staff's experiences of pain assessment in the EoL with the hope of improving care for those who are not able to verbalize pain at the EoL. Therefore, the aim of this study was to describe staff's reflections on aspects influencing pain assessment in EoL care in nursing homes before and after the implementation of a systematic pain assessment scale, the Abbey Pain Scale – APS.

## 3 THE STUDY

### 3.1 Design and setting

A retrospective qualitative approach was used. Focus group interviews were conducted with staff in specific local nursing homes regarding their experiences of pain assessment in the EoL care of people with cognitive impairment before and after implementation of the Abbey Pain Scale (APS) as a tool for systematic pain assessment. Data were obtained from three selected nursing homes in a medium‐sized city in northern Sweden with a staff/resident ratio of about 0, 95 (Harrington et al.., 2012). The selected nursing homes were of great size, having 48–110 residents with cognitive impairment (many with dementia but not all diagnosed) and most residents required day and night care until the end of life.

### 3.2 Methods

#### 3.2.1 The instrument

The APS was used as a systematic pain assessment since it was recommended as a suitable instrument for people with dementia and cognitive impairment and was translated into Swedish (RCC, 2018; The Swedish Palliative register, 2018). The instrument has earlier been tested for psychometrics properties showing Cronbach's alpha of 0.81, demonstrating a high degree of reliability, and a Gamma of 0.586, demonstrating a reasonable degree of validity (Abbey et al., 2004). The version referred to here is now undergoing the psychometric testing in a Swedish context. The Swedish version of the APS (The Swedish Palliative register, 2018) concerns six areas: changed vocal expression, changes in facial expressions, changes in body language, behaviour and physiological (changes in heart rate, blood pressure) and physical changes (contractures, ulcers, etc.). Before the introduction of the APS, a guide for use was developed in collaboration between the municipality's medical responsible nurse and the Swedish Palliative Register. The guide described that it would be used before and after the symptom relief and always at the onset of pain for those with cognitive impairment.

#### 3.2.2 Participants

The unit managers were asked to select staff who were on duty and had at least 6 months of work experience of taking care of people with cognitive impairment to participate in the study. Before data collection started, the unit managers and the nurses at the selected lodgings were informed by email about the purpose of the working process and the introduction of APS. To ascertain the variety of staff's diverse perspectives of pain assessment, four focus groups, including 4–7 informants, were conducted. The participants consisted of 11 registered nurses, 19 enrolled nurses, two assistant nurses and three care staff members who had no medical education. All participants had 2–39 years of experience working in care of the elders and had been employed in the municipality from 1.5–29 years.

#### 3.2.3 Data collection

One session for each focus group was planned both before and after the introduction of the APS. Eight focus group interview sessions (FGD) were held, which lasted from about 38–66 min (median = 53). The first sessions were conducted between September and October 2012 and the second from September to November 2013. The FGDs were open but had a discussion character. Participants were asked to relate their experiences and systematic use of pain assessment among people with cognitive impairment in EoL care. One moderator and one observer were present during the interviews.

Enrolled and assistant nurses at the selected nursing homes were interviewed individually at their workplace. The registered nurses were interviewed in one group since they had a consultant role and worked at several different units. The interviews were semi‐structured and the questions and conversation followed the participants' answers. Questions covering specific thematic areas were asked in all interviews but not always in the same order. The first interview began with the question “Can you tell about your experiences and thoughts about the use of instruments for pain assessment when taking care of people at the end of life”? Follow‐up questions focused on experiences of assessing/detecting pain and procedures in their units regarding pain assessment as well as their reflections on difficulties and challenges when using such instruments. Approximately 1 year after the APS was implemented, the second interview was held. This interview began with a similar question, which specifically focused on what experiences/concerns the staff had regarding the use of APS and was followed up by questions concerning their experiences and routines for pain relief and changes of care over the past year.

#### 3.2.4 Analysis

The interviews were transcribed verbatim and analysed by using qualitative content analysis (Graneheim & Lundman, 2004). Sentences or phrases relevant to the aim were formed into meaningful units, condensed, coded and formed into sub‐categories that were abstracted into categories. The analysis was conducted in constant movement back and forth between the different steps and resulted in two themes and ten categories that together reflected the staff's experiences of pain assessment before and after the implementation of APS in the nursing homes.

#### 3.2.5 Ethics

Before the study started, all unit managers and participants received written and oral information about the study and interviews. Participants had the right to withdraw from the study at any time, without giving any reasons and without any negative consequences. Information collected from the healthcare staff was kept confidential. Since this study was a part of a quality improvement project and did not involve residents, no Research Ethics Committee approval was required according to Swedish law (SFS 2003:460). However, the study followed ethical guidelines for research.

## 4 RESULTS

Two themes were identified and consisted of categories based on the staffs' experiences of knowledge, routines, feelings and attitudes to pain assessment in the EoL care among people living at the nursing homes (Table 1).

**TABLE 1:** Categories and themes about aspects of importance for the pain assessment before and after implementation of APS

Domain	Theme	Category
Before the introduction of APS	Striving to achieve control by trusting oneself and the team	Having continuity in the team Perceiving lack of experience and knowledge Putting attention on changes in symptoms and behaviours Experiencing a lack of time and clear routines Daring to be close to dying and death Dealing with uncertainty and frustration
After the introduction of APS	Improving symptom control with remaining weak confidence in the team	Gaining improved knowledge Perceiving a more confident assessment Identifying still unclear routines Experiencing a lack of trust in the team

### 4.1 Before the introduction of APS

#### 4.1.1 Striving to achieve control by trusting oneself and the team

Before the introduction of the APS, the staff pointed out how they tried to control pain and agreed that there must be prerequisites for systematic pain assessment since they wanted to feel safe when taking care of people at the EoL. The result shows that continuity in the care team together with knowledge and experiences of care and pain assessment are important due to the complexity of the assessment of the symptoms when a dying resident has difficulty in expressing pain verbally. The staff expressed that it was important to know the person well to be able to see changes in the residents' behaviour. It was frustrating if the resident did not receive pain relief and the staff expressed that they often felt insecure in the assessment of a person's condition and could have different views about it. Daring to be close to dying people and following clear care routines, along with knowledge and time for pain assessment, were desirable since presence and sensitivity were described as prerequisites for detecting symptoms of pain.

##### Having continuity in the team

Personal acquaintance with the resident was something that was deemed important for not only assessing the residents' symptoms but also for distinguishing between pain and anxiety. The staff expressed that long‐term contact and continuity in the relationship of care provided an opportunity for better knowledge of the resident's history of illness and symptom relief. This made it easier to detect, follow up and take care of any symptoms that could occur. They conveyed that they often found it difficult to assess signs of pain as well as symptoms indicating anxiety among new residents. The staff described that they had no reference frame to compare/relate the symptoms to if they did not know the resident well. The staff also expressed that they felt a sense of reliability and trust with a stable staff group, as continuity gave a sense of a more confident pain assessment:

You know them usually quite well. I mean, if you know their pain problems before, then you can relate it to that. If she has been fully mobile and had no inconvenience [earlier], yes, then maybe there is something else.

##### Perceiving lack of experience and knowledge

The staff expressed that pain assessment was difficult and demanding and that they perceived themselves in general as not good enough to assess and treat pain. Their perception was that the knowledge of symptoms of pain was inadequate and increased education about this was desired. They experienced that lack of knowledge could sometimes cause a feeling of insecurity since many times they could not have been sure whether the assessment and treatment of pain were optimal. In particular, they expressed that newly employed and inexperienced staff needed additional knowledge of symptoms of pain and pain assessment. More experienced staff related that they tried to influence the staff's work schedule to avoid having new and inexperienced staff members work alone in a ward unit due to their perceived lack of knowledge of pain and related symptoms. They described how experienced staff often felt that they had to share their knowledge regarding assessment of pain and taught new employees both by practically showing and by verbally explaining:

Yes, we all have a lack of knowledge [in pain assessment], even the doctors are insecure in dealing with pain. That creates much distress among the staff.

##### Putting attention on changes in symptoms and behaviours

To continuously control symptoms of pain, some participants expressed how they prioritized observing signs of pain, anxiety and avoidant behaviour, as well as follow up potential changes of symptoms when the resident had received drugs designed to relieve such symptoms. When a dying resident was perceived as becoming more concerned, the staff described that they tried to be more present with that person, which enabled them to get a better overview of potentially changed conditions and to detect new symptoms. The staff related that they observed the signs such as wrinkles in the forehead and sweaty glossy skin, if the resident felt stressed and worried, as possible symptoms of pain:

But maybe there's something new that you observe […] usually, there are several things that you possibly may notice […] or that there will be a small change in the person's behaviour or the way they are making noises.

##### Experiencing a lack of time and clear routines

The staff described that they lacked knowledge of or noticed any written routines for systematic pain assessment in general, specifically in the EoL care. The participants described how they instead tried to trust each other to continuously observe and follow up the dying residents' general condition and any changes. The participants stressed that it was important to allocate time to be able to make pain assessment, something perceived as much easier to perform during day hours when more people were working. During night hours, the staff described situations where they did not have enough time to make an adequate pain assessment, which could lead to a delay in symptom relief. Some of the staff described situations when time and clear routines were lacking to help them assess the overall picture of the residents' condition when working alone. In these situations, they usually trusted each other's skills about symptom assessment:

We are running out and in [to the resident], but we do not have or use any paper [assessment scales] to follow then.

##### Daring to be close to dying and death

To provide the best care at the EoL, the staff expressed that it was important to feel secure and not afraid of death and dying people. They expressed that there were colleagues who were anxious and fearful of death and did not dare to enter a dying person's room. The nursing staff perceived that on such occasions, the dying residents could be at risk of not being continuously observed and assessed for symptoms and thereby not receive the best care, something which frustrated the staff. Hence, they thought about the impact of their view of death and dying in their care and, consequently, pain assessment for the resident in the EoL:

And then it depends on how you experience and think about death. I mean, some think it's hard to walk in [to a dying person], even though you may have been working for 30 years, but still, do not want to be confronted with it.

##### Dealing with uncertainty and frustration

The staff expressed uncertainty regarding pain assessment when taking care of residents at EoL. They described how symptoms of pain were challenging to assess, at the same time as they expressed it beneficial to have experience and knowledge of symptoms related to pain and that they had to trust their knowledge. The staff described that they had to use skills to visually assess and interpret observed bodily symptoms and changed behaviour since the dying residents could not express themselves verbally. They described it as challenging to determine whether a specific symptom was pain, anxiety or both. They described that pain could be shown as anxiety in the same way that anxiety could indicate pain or discomfort. The responsible nurses also described challenges and uncertainty regarding the assessment of pain and symptom treatment since they mostly acted as a consultant. They described how they felt alone in their assessment and therefore were not always sure that their assessment resulted in optimal symptom relief for the residents. They expressed that they became frustrated when being consulted about assessments of pain relief and prescriptions of individual drugs as needed (Pro re‐nata) and felt that support from the team colleagues was inadequate. Furthermore, enrolled nurses and nursing assistants described feelings of frustration and insufficiency since they experienced that although they had observed and reported symptoms interpreted as pain, the nurse did not always dare to give the dose that was optimal, based on the needs for the residents:

I think she was quiet maybe two hours and then she shouted again. If it had been my mother, then I would have called for some sedative and Morphine so that she could have peace and not lying there screaming.

### 4.2 After the introduction of APS

#### 4.2.1 Improving symptom control with remaining weak confidence in the team

After the introduction of systematic pain assessment by using APS, the staff experienced that they had improved their knowledge and became better in detecting and assessing symptoms of pain. Although the staff expressed that systematic use of APS resulted in a better symptom assessment, they still expressed that unclear routines and the lack of trust in the team had an impact on the assessment of pain‐related symptoms.

##### Gaining improved knowledge

The staff expressed a positive feeling based on their experiences of using a systematic pain assessment. They described that they had received a tool that they could use, partly to remind them of symptoms that could be pain, as well as for systematically following and evaluating symptoms and relief. The staff found that their knowledge of signs of pain increased in connection with the use of the APS. They also expressed that they reflected more on other additional symptoms of pain than they previously knew:

Yes, but you see and understand, maybe you become more aware when they [residents] have pain, so there is more attention in that way and you become probably more aware of pain than you were before

However, a few participants stated that the introduction of the APS was not necessary and that it was perceived as offensive that their clinical appearance and long experience were insufficient to assess symptoms of pain.

##### Perceiving a more confident assessment

The staff expressed that systematic pain assessment made it easier to discover, monitor and get a complete picture of symptoms. It was experienced that the APS helped them to feel secure in their assessment of pain and they could rely on the assessment when they followed up the need for pain relief for these people. They described that previously they were not confident nor fully knew how to assess and follow up the intensity of pain, but now they had an assessment tool – they went from assuming to knowing. In addition, they also perceived that the dying person received better and faster pain relief when a systematic pain assessment was performed. Those who had received information about the APS and how it should be used experienced that they now could substantially demonstrate the resident's pain. Therefore, they expressed that the scale facilitated that signs of pain were not forgotten:

I have done an assessment and I … scored the pain to 12. I think we might have to do something [about the pain]. It becomes more concrete when you have something to show directly to your nurse as well.

##### Identifying still unclear routines

The staff described that the introduction of systematic pain estimation was time‐consuming. They reported that before as well as after the introduction, written procedures lacked not only systematic pain assessment but also generally the care in the EoL. The staff stressed the need for clear routines and information about the APS, especially as it was described that not all the staff had knowledge or information about the decision to use the APS. Furthermore, the staff stated that it was important that the nurse initiated the estimation of symptoms with the scale and that education about the scale should be offered to all staff in the team. It was expressed that pain assessment was found to be important but difficult and therefore clear procedures for systematic pain assessment regarding frequency, reporting, follow‐up and documentation were requested. Such routines were perceived and expected to facilitate and improve systematic pain estimation – something they experienced was forgotten due to lack of clear routines:

But I do not have … I have not received any information. Suddenly we were told to use this. I have no idea, okay, I just read and follow.

##### Experiencing a lack of trust in the team

The nurses related that doctors sometimes did not always trust the nurse's pain assessment. They also expressed frustration about not being listened to about anything that was perceived to lead to inadequate pain relief for residents. The nurses described how they often felt that physicians' prescriptions did not follow the usual recommended symptomatic palliative ordinances despite their communication and information. They expressed that in such situations, they were recommended by physicians to call back again if they perceived that given ordinances of symptomatic medication did not produce the desired effect. This was experienced as making optimal symptom relief difficult for the nurses:

Some [doctors] do not trust our skills.

Similarly, enrolled and assistant nurses experienced – in the same way as nurses did to doctors – a lack of trust and weak communication and cooperation in the team. They described that they were not always trusted or listened to by registered nurses regarding their assessment of symptoms, which sometimes resulted in suboptimal symptom relief. Additionally, they expressed that there was a lack of adequate information about pain assessment and how it would be evaluated when taking care of residents in the EoL:

The nurses that are here; they are many times more questioning, they check and make their own decisions and do not listen so much what we have to say

## 5 DISCUSSION

The results showed that before the introduction of APS, the staff stressed the importance of continuity in the team, knowledge of systematic pain assessment and clear routines. Moreover, they dare being close to dying and dealing with uncertainty and frustration when trying to achieve trust in each other and control of pain. After the introduction of APS, staff experienced that pain control was improved, by gaining knowledge and confidence in pain assessment. However, there were still unclear routines and some experienced a lack of trust in the team – obstacles of importance to overcome.

### 5.1 Before the introduction of APS

Distinguishing changes in behaviour and symptoms were described as particularly difficult if the staff lacked knowledge of the residents and their history of illness. Similarly, other studies show that knowledge of the person's background facilitates pain assessment and improves symptom relief (Brorson et al., 2014; Cunnigham et al., 2010; Krumm, Larkin, Connolly, Rode, & Elsner, 2014; McAuliffe et al., 2009; Tsai et al., 2018). The participants in this study expressed how they experienced difficulties in assessing symptoms of pain among new residents at the nursing home since there lacked frames of reference. The staff perceived long‐term contact between them, and the residents meant continuity, which helped them to assess pain. Brorson et al. (2014) discuss the challenge of assessing pain in palliative care since difficulties in expression and communication are common among people with dementia, which necessitates long‐term contact to recognize behavioural changes and to detect pain.

Lack of knowledge was experienced as a reason for poorer systematic assessment and relief of pain. Furthermore, the staff expressed a need for additional education and training, similarly as others (Gropelli & Sharer, 2013; Kaasalainen et al., 2013; Tousignant‐Laflamme et al., 2012), emphasizing that education should be prioritized to decrease emphasizing suffering. It is essential that all healthcare staff have sufficient knowledge of assessment and treatment of pain to apply good care at the EoL (Kaasalainen et al., 2013; van der Steen, 2010; van der Steen et al., 2014; The National Board of Health & Welfar [Socialstyrelsen], 2013a). Improved knowledge of the subject is required (Burns & McIlfatrick, 2015; van der Steen et al., 2014; Tarter et al., 2016) since the most important components of care in EoL are timely detection, good assessment and relief of pain.

The results show that staff lacked written routines and time for assessing pain, although guidelines for palliative care emphasize the necessity of good and well‐established routines (Burns & McIlfatrick, 2015; van der Steen et al., 2014; The National Board of Health & Welfar [Socialstyrelsen], 2013a, 2013b). In line with our study, McConigley, Toye, Goucke, and Kristjanson (2008) and others (Burns & McIlfatrick, 2015; Sampson et al., 2015) indicate that guidelines and education and training that focuses on assessment and relief of pain in EoL care are necessary when taking care of people with dementia and cognitive impairment. Additionally, having time to perform systematic pain assessment appears to lead to improved symptom relief for the care recipient while the staff are less stressed (Burns & McIlfatrick, 2015; Fuchs‐Lacelle et al., 2008).

The result indicates that staff working closest to dying residents described a frustration since they perceived that pharmacological pain relief was not always adequate even though they observed and reported clear signs of pain. The complexity of recognizing and treating pain adequately was previously described (Tarter et al., 2016). McAuliffe et al. (2009) found that changed behaviour related to pain is commonly interpreted as psychosomatic and therefore not primarily treated with pain relievers. Difficulties in identifying pain may, therefore, contribute to inadequate pain relief (Tsai et al., 2018).

### 5.2 Following the introduction of APS

The participants in our study described improved knowledge and an increased sense of confidence in pain assessment after the introduction of APS. They stated that using the APS helped them develop a new viewpoint about symptoms of pain. Others also described an increased awareness of multiple symptoms and clarity of what to assess. Krumm et al. (2014) use the word “expanding horizons,” describing the experience of the staff in connection with the introduction of a taxation instrument for palliative care for people with dementia. Consistent with others (Abbey et al., 2004, Ni Thuathail & Welford, 2011), the APS was experienced as a simple, time‐saving assessment scale useful for detecting pain and evaluating treatment.

The staff in our study also experienced that with improved knowledge that facilitated pain assessment they could more easily recognize if a dying person required pain relief. Using instruments for systematic pain assessment also enabled them to better grasp a picture of symptoms and changes over time. In accordance with the national recommendation and literature (Burns & McIlfatrick, 2015; The National Board of Health & Welfar [Socialstyrelsen], 2013a), systematic assessment and analysis of symptoms are required to detect and treat pain at the EoL. A previous study (Liu, 2014) described that nurses working close to a dying person benefit greatly from systematic assessment in identifying symptoms related to pain as early as possible.

Our results show that despite information about the use of the APS, routines about pain relief in EoL care were lacking. The staff experienced that pain assessment was difficult and assessment using APS was often forgotten since such situations did not occur every day. The staff asked for more education and training as well as access to written procedures. They also pointed out that it was important that a nurse could initiate pain relief. Van der Steen (2010) and van der Steen et al. (2014) point to the importance of clear guidelines for nursing and treatment and that pain assessment tools are regularly used to enable a safe assessment of pain.

The lack of communication and trust in the care team was found to result in inadequate assessments and insufficient pain relief as well as staff frustration. Similarly, Brorson et al. (2014) describe that nurses do not feel that physicians trusted their pain assessment. This is confirmed in a Swedish study (Dwyer, Hansebo, Andershed, & Ternestedt, 2011) where the staff expressed that nurses and physicians do not know the resident well enough to prescribe correct pain relief. This indicates the importance of well‐functioning cooperation and trust in the team for good EoL care. The importance of good communication and well‐functioning teamwork is also emphasized by others (Burns & McIlfatrick, 2015, Cunnigham et al., 2010, Gropelli & Sharer, 2013, Krumm et al., 2014, Liu, 2014, Newton et al., 2014).

### 5.3 Strengths and limitations

This study is performed in a specific context which might have impact on the results and its transferability. However, our results are based on the experiences of nursing staff both before and after implementation of APS and highlighted the importance of good collaboration and trust in the team when taking care of residents who are in the EoL. A limitation of this study could be the recruitment of participants. Additionally, we have not observed the situations of pain assessment; however, the existence of multi‐professionality including the follow‐up interviews and the great variation of experiences are decisive points in this study.

## 6 CONCLUSION

It was felt that the use of APS as a systematic pain assessment can lead to better symptom and pain management among people with cognitive impairment at EoL. However, knowing the resident well and trusting the team facilitate proper pain assessment. To make adequate assessments of pain at EoL, the staff needs more training in systematic pain assessment and attentiveness to new symptoms and changes in behaviour that may be signs of pain. Therefore, although a systematic use of APS may improve assessment and treatment of pain and symptoms in EoL, a lack of confidence in the care team may jeopardize optimal pain assessment and pain relief in EoL care. Furthermore, to maintain the competence in the area and a good quality in EoL care, unit managers have an important role to prioritize, support and further develop a feeling of unity and respect as well as improve communication among team members.

## CONFLICT OF INTEREST

The authors have no conflict of interest to declare.

## AUTHOR CONTRIBUTIONS

CL and SH: Study design and data collection. CL, SH and UI: Analysis and data interpretation. All authors have been drafting, revising and preparing the article to the submission. The final version was read and approved by all authors.

## References

[nop2566-bib-0001] Abbey, J. , Piller, N. , De Bellis, A. , Esterman, A. , Parker, D. , Giles, L. , & Lowacy, B. (2004). The Abbey pain scale: A 1‐minute numerical indicator for people with endstage dementia. International Journal of Palliative Nursing, 10, 6–13. 10.12968/ijpn.2004.10.1.12013 10.12968/ijpn.2004.10.1.12013 14966439

[nop2566-bib-0002] Afzal, N. , Buhagiar, K. , Flood, J. , & Cosgrave, M. (2010). Quality of end‐of‐life care for dementia patients during acute hospital admission: A retrospective study in Ireland. General Hospital Psychiatry, 32, 141–146. 10.1016/j.genhosppsych.2009.10.003 20302987

[nop2566-bib-0003] Brorson, H. , Plymoth, H. , Örmon, K. , & Bolmsjö, I. (2014). Pain relief at the end of life: Nurses' experiences regarding end‐of‐life pain relief in patients with dementia. Pain Management Nursing, 15, 315–323. 10.1016/j.pmn.2012.10.005 23453467

[nop2566-bib-0004] Burns, M. , & McIlfatrick, S. (2015). Palliative care in dementia: Literature review of nurses' knowledge and attitudes towards pain assessment. International Journal of Palliative Nursing, 21, 400–407. 10.12968/ijpn.2015.21.8.400 26312536

[nop2566-bib-0005] Cunnigham, C. , McClean, W. , & Kelly, F. (2010). The assessment and management of pain in people with dementia in care homes. Nursing Older People, 22, 29–35. 10.7748/nop2010.09.22.7.29.c7947 20882781

[nop2566-bib-0006] Dwyer, L. L. , Hansebo, G. , Andershed, B. , & Ternestedt, B.‐M. (2011). Nursing home residents' views on dying and death: Nursing home employee's perspective. International Journal of Older People Nursing, 6, 251–260. 10.1111/j.1748-3743.2010.00237.x 21631874

[nop2566-bib-0007] Fuchs‐Lacelle, S. , Hadjistavropoilos, T. , & Lix, L. (2008). Pain assessment as intervention: A study of older adults with severe dementia. Clinical Journal of Pain, 24, 697–707. 10.1097/AJP.0b013e318172625a 18806535

[nop2566-bib-0008] Fürst, C. J. , Lindqvist, O. , & Tishelman, C. (2012). Towards a basic drug kit for the dying patient. Current Opinion in Supportive and Palliative Care, 6, 386–390. 10.1097/SPC.0b013e328356ab5e 22801469

[nop2566-bib-0009] Gilmore‐Bykovskyi, A. , & Bowers, B. (2013). Understanding nurses decisions to treat pain in nursing home residents with dementia. Research in Gerontological Nursing, 6, 127–138. 10.3928/19404921-20130110-02 23330944 PMC3634876

[nop2566-bib-0010] Givard, K. , & Poulsen, I. (2013). Implementering af en smerte med nedsat bevidsthed. Sygeplejersken, 11, 66–72

[nop2566-bib-0011] Graneheim, U. H. , & Lundman, B. (2004). Qualitative content analysis in nursing research: Concepts, procedures and measures to achieve trustworthiness. Nurse Education Today, 24, 105–112. 10.1016/j.nedt.2003.10.001 14769454

[nop2566-bib-0012] Gropelli, T. , & Sharer, J. (2013). Nurses' Perceptions of pain management in older adults. Medsurg Nursing, 22, 375–382. 24600934

[nop2566-bib-0014] Harrington, C. , Choiniere, J. , Goldmann, M. , Jacobsen, F. F. , Lloyd, L. , McGregor, M. , … Szebehely, M. (2012). Nursing home staffing standards and staffing levels in six countries. Journal of Nursing Scholarship, 44, 88–98. 10.1111/j.1547-5069.2011.01430.x 22340814

[nop2566-bib-0015] Herr, K. , Coyne, P. J. , Key, T. , Manworren, R. , McCaffery, M. , Merkel, S. , … Wild, L. (2006). Pain assessment in the nonverbal patient: Position statement with clinical practice recommendations. Pain Management Nursing, 7, 44–52. 10.1016/j.pmn.2006.02.003 16730317

[nop2566-bib-0016] Herr, K. , & Ersek, M. (2009). Measurement of pain and other symptoms in the cognitively impaired In Hanks G. E. A. (Ed.), Oxford textbook of palliative medicine, (pp. 462–475). Oxford, UK: Oxford University Press.

[nop2566-bib-0017] Jansen, B. D. W. , Brazil, K. , Passmore, P. , Buchanan, H. , Maxwell, D. , McIlfatrick, S. J. , … Parsons, C. (2017). Exploring healthcare assistants' role and experience in pain assessment and management for people with advanced dementia towards the end of life: A qualitative study. BMC Palliative Care, 16, 6 10.1186/s12904-017-0184-1 28103847 PMC5247820

[nop2566-bib-0018] Kaasalainen, S. , Ploeg, J. , McAiney, C. , Schindel Martin, L. , Donald, F. , Martin‐Miseer, R. , … Sangster‐Gormley, E. (2013). Role of the nurse practitioner in providing palliative care in long‐term care homes. International Journal of Palliative Nursing, 19, 477–485. 10.12968/ijpn.2013.19.10.477 24162278

[nop2566-bib-0019] Krumm, N. , Larkin, P. , Connolly, M. , Rode, P. , & Elsner, F. (2014). Improving dementia care in nursing homes: Experiences with a palliative care symptom‐assessment tool (MIDOS). International Journal of Palliative Nursing, 20, 187–192. 10.12968/ijpn.2014.20.4.187 24763327

[nop2566-bib-0020] Lints‐Martindale, A. C. , Hadjistavropulos, T. , Lix, L. M. , & Thorpe, L. (2012). A comparative investigation of observational pain assessment tools for older adults with dementia. The Clinical Journal of Pain, 28, 226–237. 10.1097/AJP.0b013e3182290d90 21904200

[nop2566-bib-0021] Liu, J. Y. (2014). Exploring nursing assistants' roles in the process of pain management for cognitively impaired nursing home residents: A qualitative study. Journal of Advanced Nursing, 70, 1065–1077. 10.1111/jan.12259 24102751

[nop2566-bib-0022] Lovenheim, H. , Sandman, P. O. , Kallin, K. , Karlsson, S. , & Gustafson, Y. (2008). Symptoms of mental health and psychotropic drug use among old people in geriatric care, changes between 1982 and 2000. International Journal of Geriatric Psychiatry, 23, 289–294. 10.1002/gps.1876 17645282

[nop2566-bib-0023] McAuliffe, L. , Nay, R. , O'Donell, M. , & Fetherstonhaugh, D. (2009). Pain assessment in older people with dementia: Literature review. Journal of Advanced Nursing, 65, 2–10. 10.1111/j.1365-2648.2008.04861.x 19016920

[nop2566-bib-0024] McConigley, R. , Toye, C. , Goucke, R. , & Kristjanson, L. J. (2008). Developing recommendations for implementing the Australian Pain Society's pain management strategies in residential aged care. Australasian Journal on Ageing, 27, 45–49. 10.1111/j.1741-6612.2007.00266.x 18713216

[nop2566-bib-0025] Monroe, T. , Carte, M. , Feldt, K. , Tolley, B. , & Cowan, R. L. (2012). Assessing advanced cancer pain in older adults with dementia at the end‐of‐life. Journal of Advanced Nursing, 68, 2070–2078. 10.1111/j.1365-2648.2011.05929.x 22272816 PMC5726999

[nop2566-bib-0026] Newton, P. , Reeves, R. , West, E. , & Schofield, P. (2014). Patient‐centred assessment and management of pain for older adults with dementia in care home and acute settings. Reviews in Clinical Gerontology, 24, 139–144. 10.1017/S0959259814000057

[nop2566-bib-0027] Ni Thuathail, A. , & Welford, C. (2011). Pain assessment tools for older people with cognitive impairment. Nursing Standard, 26, 39–46. 10.7748/ns2011.10.26.6.39.c8756 22046926

[nop2566-bib-0028] RCC (2018). [Regional cancer centrum] Nationellt vårdprogram pallaitv vård [Online]. Retrieved from https://www.cancercentrum.se/norr/vara‐uppdrag/palliativ‐vard/vardprogram/

[nop2566-bib-0029] Sampson, E. L. , van der Steen, J. T. , Pautex, S. , Svartzman, P. , Sacchi, V. , Van den Block, L. , & Van Den Noortgate, N. (2015). European palliative care guidelines: How well do they meet the needs of people with impaired cognition? BMJ Supportive & Palliative Care, 5, 301–305. 10.1136/bmjspcare-2014-000813 25869811

[nop2566-bib-0030] SCB (2018). [Statistics Sweden] Störst folkökning att vänta bland de öldsta [online]. Retrieved from https://www.scb.se/hitta‐statistik/statistik‐efter‐amne/befolkning/befolkningsframskrivningar/befolkningsframskrivningar/pong/statistiknyhet/sveriges‐framtida‐befolking‐20182070/

[nop2566-bib-0031] Schulman‐Green, D. , Cherlin, E. J. , McCorkle, R. , Carlson, M. D. , Pace, K. B. , Neigh, J. , … Bradley, E. H. (2010). Benefits and challenges in use of a standardized symptom assessment instrument in hospice. Journal of Palliative Medicine, 13, 155–159. 10.1089/jpm.2009.0245 19827961 PMC2883490

[nop2566-bib-0032] SFS 2003:460. Lag (2003:460) om etikprövning av forskning som avser människor (The Swedish Law on ethical review of research concerning people) [Online]. Retrieved from https://www.riksdagen.se/sv/dokument‐lagar/dokument/svensk‐forfattningssamling/lag‐2003460‐om‐etikprovning‐av‐forskning‐som_sfs‐2003‐460

[nop2566-bib-0033] Shega, J. W. , Hougham, G. W. , Stocking, C. B. , Cox‐Hayley, D. , & Sachs, G. A. (2008). Patients dying with dementia: Experience at the end of life and impact of hospice care. Journal of Pain and Symptom Management, 35, 499–507. 10.1016/j.jpainsymman.2007.06.011 18261878

[nop2566-bib-0034] Tapp, D. , Chenacher, S. , Gérard, N. P. A. , Bérubé‐Mercier, P. , Gelinas, C. , Douville, F. , & Desbiens, J. F. (2019). Observational pain assessment instruments for use with nonverbal patients at the e‐of‐life: A systematic review. Journal of Palliative Care, 34, 255–266. 10.1177/0825859718816073 30638134

[nop2566-bib-0035] Tarter, R. , Demiris, G. , Pike, K. , Washington, K. , & Parker Oliver, D. (2016). Pain in hospice patients with dementia: The informal caregiver experience. American Journal of Alzheimer's Disease & Other Dementias®, 31, 524–529. 10.1177/1533317516653825 PMC4982799 27303062

[nop2566-bib-0036] The National Board of Health and Welfare [Socialstyrelsen] (2013a). Nationellt kunskapsstöd för god palliativ vård i livets slutskede ‐ Vetenskapligt underlag, Rekommendationer för god palliativ vård i livets slutskede; Bilaga. Retrieved from http://www.socialstyrelsen.se

[nop2566-bib-0037] The National Board of Health and Welfare [Socialstyrelsen] (2013b). Nationellt kunskapsstöd för god palliativ vård i livets slutskede – Vägledning, rekommendationer och indikatorer – Stöd för styrning och ledning. Retrieved from https://www.socialstyrelsen.se/regler‐och‐riktlinjer/nationella‐riktlinjer/publicerade‐riktlinjer/palliativ‐vard/

[nop2566-bib-0038] The Swedish Palliative register [Svenska Palliativa registret] . (2018). Årsrapport för Svenska Palliativregistret – verksamhetsåret 2013 [Online]. palliativ.se: Svenska Palliativa registret. Retrieved from https://palliativ.se/arkiv‐2/nyhetsarkiv‐2/arsrapporter/https://palliativ.se/arkiv‐2/nyhetsarkiv‐2/arsrapporter/

[nop2566-bib-0039] Tousignant‐Laflamme, Y. , Tousignant, M. , Lussier, D. , Lebel, P. , Savoie, M. , Lalonde, L. , & Choiniére, M. (2012). Educational needs of health care providers working in long‐term care facilities with regard to pain management. Pain Research & Management, 17, 341–346. 10.1155/2012/506352 23061085 PMC3465095

[nop2566-bib-0040] Tsai, I. P. , Jeong, S. Y. , & Hunter, S. (2018). Pain assessment and management for older patients with dementia in hospitals: An integrative literature review. Pain Management Nursing, 19, 54–71. 10.1016/j.pmn.2017.10.001 29153920

[nop2566-bib-0041] van der Steen, J. T. (2010). Dying with dementia: What we know after more than a decade of research. Journal of Alzheimer's Disease, 22, 37–55. 10.3233/JAD-2010-100744 20847433

[nop2566-bib-0042] van der Steen, J. T. , Radbruch, L. , Hertogh, C. M. , de Boer, M. E. , Hughes, J. C. , Larkin, P. , … Volicer, L. (2014). White paper defining optimal palliative care in older people with dementia: A Delphi study and recommendations from the European association for palliative care (EAPC). Palliative Medicine, 28, 197–209. 10.1177/0269216313493685 23828874

[nop2566-bib-0043] Zwakhalen, S. M. , Hamers, J. P. , & Berger, M. P. (2007). Improving the clinical usefulness of a behavioural pain scale for older people with dementia. Journal of Advanced Nursing, 58, 493–502. 10.1111/j.1365-2648.2007.04255.x 17442027

[nop2566-bib-0044] Zwakhalen, S. M. , Hamers, J. P. , Peijnenburg, R. H. , & Berger, M. P. (2007). Nursing staff knowledge and beliefs about pain in elderly nursing home residents with dementia. Pain Research & Management, 12, 177–184. 10.1155/2007/518484 17717609 PMC2670708

